# Anti-inflammatory activities of two new deoxygenated N-acetyl glucosamines in lipopolysaccharide-activated mouse macrophage RAW264.7 cells

**DOI:** 10.1016/j.heliyon.2023.e15769

**Published:** 2023-04-25

**Authors:** Quang Le, Zhichang Zhang, Daniel Sun, Quanjun Cui, Xinlin Yang, Ameer E. Hassan

**Affiliations:** a Dept of Orthopaedic Surgery, University of Virginia, 22903, USA; b Dept of Orthopaedic Surgery, The First Affiliated Hospital of Xinxiang Medical University, Wehui, 453100, Henan, China; c Department of Neuroscience, Valley Baptist Medical Center, 78550, USA

**Keywords:** Anti-inflammation, Glucosamine, Osteoarthritis

## Abstract

**Background:**

Glucosamine and N-acetyl-glucosamine (NAG) are amino sugars found in human extracellular matrix with previously described anti-inflammatory effects. Despite mixed results from clinical studies, these molecules have been used extensively in supplements.

**Objective:**

We investigated the anti-inflammatory properties of two synthesized derivatives of N-acetyl-glucosamine (NAG), bi-deoxy-N-acetyl-glucosamine (BNAG) 1 and 2.

**Methods:**

Using mouse macrophage RAW 264.7 cells with lipopolysaccharide (LPS) to induce inflammation, the effects of NAG, BNAG 1, and BNAG 2 on the expression of IL-6, IL-1β, inducible nitric oxide synthase (iNOS) and COX-2 were studied using ELISA, Western blot and quantitative RT-PCR. Cell toxicity and nitric oxide (NO) production were evaluated using WST-1 assay and the Griess reagent, respectively.

**Results:**

Among the three tested compounds, BNAG1 shows the highest inhibition of iNOS, IL-6, TNF α and IL-1β expression and NO production. All three tested compounds show slight inhibition on cell proliferation of RAW 264.7 cells, except that BNAG1 displays a remarkable toxicity at the tested maximum dose of 5 mM.

**Conclusion:**

BNAG 1 and 2 exhibit notable anti-inflammatory effects compared to the parent NAG molecule.

## 1 Introduction

Inflammation is an exceedingly complex physiological pathway that underlies many human pathologies [1]. Understandably, a lot of effort within the scientific community has been directed at developing new and innovative ways to attenuate the effects of pathologically hyperactive immune responses [[2], [3], [4]]. Among these, glucosamine and its derivatives are enticing choices.

Glucosamine (GlcN), also known by its IUPAC name 2-amino-2-deoxy-d-glucose, along with its acetylated product N-acetyl glucosamine (NAG) are naturally occurring amino sugars found in human extracellular matrix and the synthesis of glycosylated proteins and lipids [5]. Despite mixed and controversial results from clinical studies, animal studies have provided reasonable support to the claim that GlcN possesses anti-inflammatory properties in the management of osteoarthritis [6,7]. Nöt et al. also found that O-linked beta-NAG, a metabolic product of GlcN, is associated with attenuation of trauma-hemorrhage-related inflammatory response [8]. Hirata et al. found that the same substance suppresses acute inflammation resulting in reduced inflammation-mediated colon carcinogenesis [9]. Multiple synthetic glucosamine derivatives have also been noted for their protective/regenerative effect on cartilage [[10], [11], [12]], and anti-inflammatory properties [13]. More recently, Hassan et al. reported outcomes from a prospective study using NAG on COVID-19 patients with notable clinical benefits [14].

The current understanding of GlcN's mechanism of action is centered around its interaction with the ubiquitous NFkB pathway. Per Largo et al. GlcN suppresses the degradation of the IkBa and the migration of p50 and p65 subunits to the nucleus. As the result, GlcN prevents NFkB activation as well as downstream molecules such as COX-2 and Prostaglandin E2 [15]. Jeong et al. provided an additional mechanism, which includes GlcN binding to transglutaminase 2 and preventing IkB polymerization as well as the resulting NFkB activation [16]. Another report has recognized that GlcN also might suppress inflammation through lessened production of nitric oxide (NO) by inducible NO synthase (iNOS) [17].

**Fig. 1:**
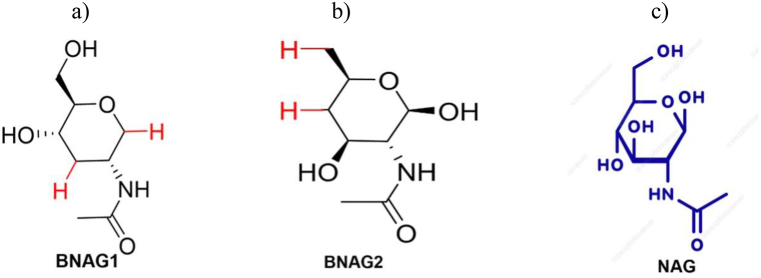
Chemical structures of BNAG1, BNAG2, and NAG. a) BNAG1, 1,3-bideoxy-n-acetyl-d-glucosamine or 2-Acetamido-1,2,3-trideoxy-d-glucose; b) BNAG2, 4,6-bideoxy-n-acetyl-d-glucosamine or 2-Acetamido-2,4,6-trideoxy-d-glucose; c) NAG, N-Acetyl-d-glucosamine or 2-Acetamido-2-deoxy-d-glucose.

As noted earlier, while GlcN's effects have been well documented in cell and animal studies, clinical results have been disappointing. As such, there have been interests in developing GlcN analogs as therapeutic options. In this study, we investigate two newly synthesized deoxygenated N-acetyl glucosamines, BNAG1 and BNAG2 (Fig. 1). The BNAG1 molecule was created by deoxygenating the original NAG molecule at the first and third carbons. The BNAG2 molecule, on the other hand, was created by deoxygenating NAG at the fourth and sixth carbons. These molecules are synthesized from NAG through multi-step processes. The resulting substance is stable at room temperature in powder form. Their protection against inflammation of lipopolysaccharide (LPS)-activated mouse macrophage RAW264.7 cells was assessed, using N-acetyl glucosamine (NAG) as a control. To evaluate the anti-inflammatory effects of BNAG 1 and 2, we evaluate their influence on the production of classical inflammatory markers IL-6 and IL-1β. Moreover, we also evaluate the markers NO and COX-2, both of which have been implicated in GlcN's effect pathway.

## 2 Materials and methods

### 2.1 Synthesis of BNAG1 and BNAG2

BNAG1 (purity>95%) was synthesized from NAG through a 7-step procedure. For BNAG2 synthesis, the starting material was NAG (Catalog No. ICN19460405, Thermo Fisher Scientific, Pittsburgh, PA, USA) with protected 1, 4, 6-hydroxyl groups, and BNAG2 (purity>95%) was obtained after 6 chemical reactions. BNAG1: LC-MS: 190.20 [M+1]^+^. ^1^H NMR (400 MHz, CD3OD) δ 3.94–3.79 (m, 3H), 3.61–3.52 (m, 1H), 3.48–3.41 (m, 1H), 3.05–2.95 (m, 2H), 2.25–2.18 (m, 1H), 1.90 (s, 3H), 1.37 (q, *J* = 11.7 Hz, 1H). BNAG2: LCMS: 190.20 [M+H]^+^. ^1^H NMR (400 MHz, DMSO‑*d*_6_): δ 7.55 (s, 1 H), 6.24 (s, 1 H), 4.89 (s, 1 H), 4.54 (s, 1 H), 3.98–3.95 (m, 1 H), 3.66–3.62 (m, 1 H), 3.45–3.40 (m, 1 H), 1.87–1.83 (m, 1 H), 1.79 (s, 3 H), 1.13–1.07 (m, 1 H) 1.03 (s, 3 H). The detailed synthesis of BNAG1 and BNAG2 will be reported in another article.

### 2.2 Cell treatment

Mouse RAW264.7 macrophage cell line (Catalog No. TIB-71™, purchased from ATCC; Manassas, VA, USA) was maintained in a DMEM culture medium (Catalog No. 11885084, Thermo Fisher Scientific) containing 10% FBS (Catalog No. 10082147, Thermo Fisher Scientific) and 1% penicillin-streptomycin-glutamine mix (100X) (Catalog No. 10378016, Thermo Fisher Scientific). For cell treatment, BNAG1 or BNAG2, or NAG was added to the culture for 0.5 h before the addition of 100 ng/mL LPS (Catalog No. L3024, Sigma-Aldrich Co., St Louis, MO, USA) and then cells were treated with LPS and BNAG1 or BNAG2, or NAG for 24 h. In addition, 96 well plates were used for tests for cell proliferation, nitrite production and IL6 and TNF α level, while 24 well plates and 6 well plates for gene expression and Western blot assays. The initial cell numbers of approximately 20,000, 100,000 and 500,000 per well were seeded on 96, 24 and 6 well plates, respectively. Furthermore, the doses of each drug varied with different tests and were described below.

### 2.3 Cell proliferation test

RAW264.7 cells were seeded on a 96 well plate, followed by treated with BNAG1, BNAG2 and NAG at doses of 0–5 mM for 24 h. The cell number in each well was counted using the WST-1 kit (Catalog No. 50443941, Thermo Fisher Scientific) according to the manufacturer's instructions. After removal of the supernatant, cells were incubated with 150 μL of the fresh medium along with 15 μL of the WST-1 reagent/each well for 3 h in dark at 37 °C. The optical density (OD) was determined at 450 nm on a microplate reader [18]. The inhibition rate of each test group was calculated as [OD of non-treatment (NT) – OD of test (T)]/OD of NT x 100%.

### 2.4 Nitrite production

RAW264.7 cells were seeded on a 96 well plate, followed by treated with BNAG1, BNAG2 and NAG at doses of 0–0.1 mM for 24 h. Nitrite in the culture medium was detected by a commercial Griess reagent kit (Catalog No. G2930, Promega Corporation, Madison, WI, USA), according to the manufacturer's manual. This kit utilizes the chemical reaction involving sulfanilamide and N-1-napthylethylenediamine dihydrochloride (NED) under acidic (phosphoric acid) conditions. Briefly, all experimental samples and standards containing the dilution series for the nitrite standard curve were mixed with 50 μL of the sulfanilamide solution ((1% sulfanilamide in 5% phosphoric acid) and incubated for 10 min at room temperature without light in a 96-well plate. Then 50 μL of the NED Solution (0.1% NED in water) was added to each well and incubated for 10 min again at room temperature without light. The OD value in each well at 530 nm of the absorption wavelength was immediately determined on a microplate reader [18]. Meanwhile, the cell number in each well was assessed by the WST-1 test. Then the OD value from the nitrite test was divided by the OD value from the WST-1 test to obtain the normalized nitrite amount in each well.

### 2.5 IL-6 and TNF α level

Cells were seeded on a 96-well plate and the same treatments as nitrite test were performed. The amounts of IL-6 and TNF α in the culture medium from different treatments were measured by the commercial mouse IL-6 and TNF α ELISA kits (Catalog No. KMC0061 for IL-6 and BMS607-3 for TNF α, Thermo Fisher Scientific), following the instructions provided by the manufacturer [18]. Meanwhile, the cell number in each well was assessed by the WST-1 test. Then the OD value from the IL-6 and TNF α ELISA was divided by the OD value from the WST-1 test to obtain the normalized OD values of IL-6 and TNF α in each well, respectively.

### 2.6 Gene expression

Cells were seeded on a 24-well plate and various treatments were performed with 0.5 mM of BNAG1, BNAG2, and NAG, respectively. Cellular RNA was purified using an RNeasy kit (Catalog No. 74104, QIAGEN Sciences, Valencia, CA), and the total RNA yield and purity were estimated using microplate reader at 260/280 nm. Synthesis of cDNA and the quantitative PCR (qPCR) was carried out using the iscript™ cDNA synthesis kit (Catalog No. 1708891) and the iQ™ SYBR Green Supermix kit (Catalog No. 1708882, Bio‐Rad Laboratories, Hercules, CA), respectively. The qPCR conditions were for pre-denaturation (95 °C for 5 min), denaturation (95 °C for 1 min), annealing (See the temperature for different target gene in Table 1) for 40 s, pre-elongation (72 °C for 1 min), and elongation (72 °C for 5 min). All the reaction was set for 55–90 °C melting curve and 4 °C infinite hold, in a total of 40 reaction cycles. The threshold cycle (CT) value was calculated from amplification plots. Data were analyzed using the 2-ΔΔCT method with 18s rRNA serving as reference [18]. Gene expression was normalized to the control group in each experiment and represented as fold change. The target genes included iNOS and IL-1β. Gene of 18s ribosomal RNA was used as an internal control. The primer sequences were provided in Table 1.

**Table 1:** The primer sequences used for the quantitative RT-PCR.

Target	Forward primer (5′-3′) (length)	Reverse primer (5′-3′) (length)	Product size (bp)	Annealing (°C)
IL-1β	CAA CCA ACA AGT GAT ATT CTC CAT G **(25 nt)**	GAT CCA CAC TCT CCA GCT GCA **(21 nt)**	152	62
iNOS	TCC TAC ACC ACA CCA AAC **(18 nt)**	CTC CAA TCT CTG CCT ATC **(18 nt)**	199	55
18s rRNA	CGG CGA CGA CCC ATT CGA AC **(20 nt)**	GAA TCG AAC CCT GAT TCC CCG TC **(23 nt)**	99	65

### 2.7 Western blot

The cellular proteins were prepared from cells growing on a 6-well plate after treatments of BNAG1, or BNAG2 or NAG using a commercial RIPA buffer (5X) (Catalog No. AAJ62524AD, Thermo Fisher Scientific) containing a protease inhibitor cocktail (Catalog No. sc-29130, Santa Cruz Biotechnology Inc., Dallas, TX, USA) and 1 mM phenylmethylsulfonyl fluoride (Catalog No. sc-482875, Santa Cruz Biotechnology Inc.). Then the protein concentration was determined using the Bradford protein assay kit (Catalog No. 5000201, Bio-Rad, Hercules, CA, USA). SDS-polyacrylamide gel electrophoresis, protein transfer to nitrocellulose membranes (Catalog No. 88025, Thermo Fisher Scientific), incubation of membranes with primary antibodies against mouse iNOS and Cox-2 (Catalog No. NB300-605 and NB110-1948, both from Novus Biologicals, LLC), and horseradish peroxidase (HRP)-conjugated secondary antibody (Catalog No. 7074S, Cell Signaling), and visualization of protein bands were performed according to the procedures described previously [18]. β-actin (Catalog No. sc-47778, Santa Cruz Biotechnology Inc.) was used as the loading control. All Western blot assays were conducted in duplicates.

### 2.8 Data analysis

**Fig. 2:**
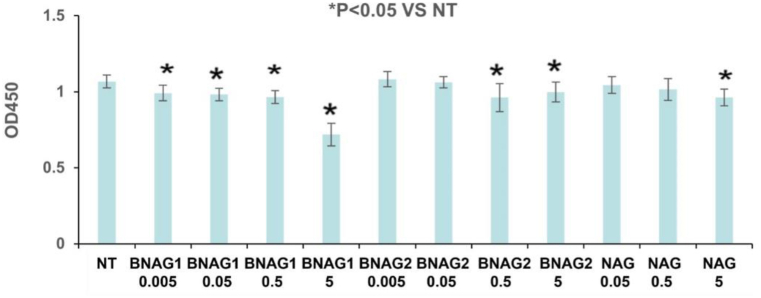
**The different effects of BNAG1, BNAG2 and NAG on cell growth of mouse macrophage cell line RAW264.7 cells**. Concentrations up to 5 mM were tested for each drug. No drugs were added to the non-treatment group (NT). There were eight repeats in each group (n = 8). *P < 0.05 vs NT group. The experiment was duplicated.

**Fig. 3:**
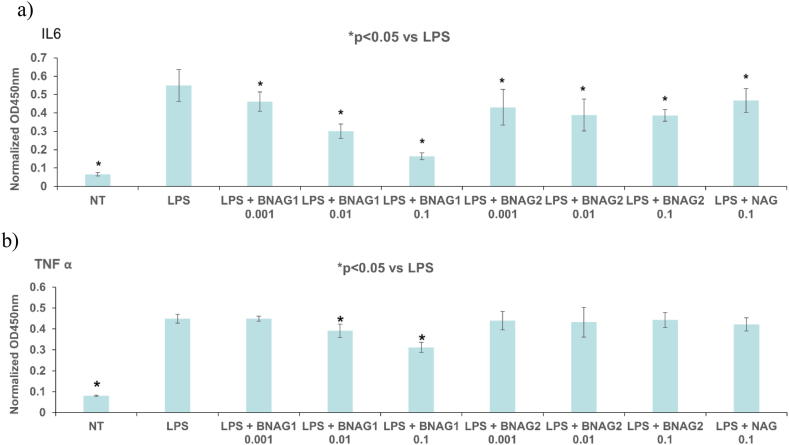
**The comparison of BNAG1, BNAG2, and NAG in IL-6 (a) and TNF α (b) production in RAW264.7 cells**. Various concentrations (0.001 mM, 0.01 mM and 0.1 mM were tested for BNAG1 and BNAG2, while NAG at 0.1 mM was used as control. LPS: 100 ng/mL. There were eight repeats in each group (n = 8). *P < 0.05 vs LPS group. The experiment was duplicated.

All data was reported as mean ± SD. A one-way ANOVA test with multiple comparisons was performed. NT group in Fig. 2 or LPS group in Fig. 3, Fig. 5, Fig. 6 was used as a control data, obtaining the p-Value parameter as result. These p-Values indicated if the difference between OD value of NT group in Fig. 2 or LPS group in Fig. 3, Fig. 5, Fig. 6 compared with the rest of the samples was significant [P-value <0.05 (*)]. The GraphPad Prims program was used to obtain the results.

## 3 Results

### 3.1 WST-1 assay for cell proliferation

The WST-1 assay showed that BNAG1 was the most toxic against mouse macrophage cell line RAW264.7 cells among the tested three drugs at the dose range of 0–5 mM. BNAG1 at all four doses (0.005 mM, 0.05 mM, 0.5 mM and 5 mM), BNAG2 at 0.5 mM and 5 mM, and NAG at 5 mM could significantly inhibit cell proliferation (p < 0.05, n = 8). Notably, the inhibition rate for each drug at every dose was less than 10% except that the inhibition rate was 32.6% for BNAG1 at 5 mM (Fig. 2), indicating that the impact of each drug on cell proliferation was relatively low at the doses no bigger than 0.5 mM.

### 3.2 ELISA test for IL-6 and TNF α production

The ELISA tests showed that all three drugs tested (BNAG1, BNAG2, and NAG) could significantly reduce IL-6 production in LPS-activated mouse RAW264.7 macrophages, while BNAG1 was most effective among them (n = 8). In addition, only BNAG1 at 0.01 mM and 0.1 mM was able to significantly reduce TNF α production (Fig. 3).

### 3.3 Western blot for iNOS and Cox-2 expression

**Fig. 4:**
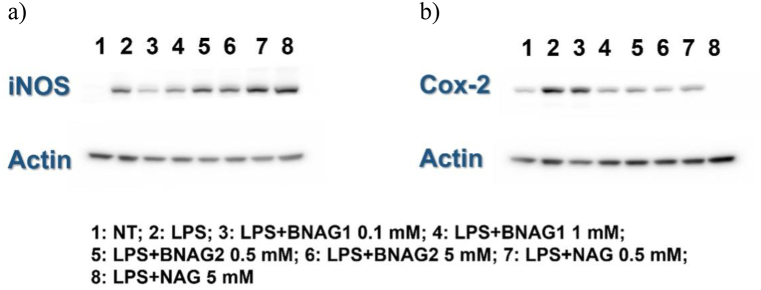
**The comparison of BNAG1, BNAG2, and NAG in iNOS (a) and Cox-2 (b) expression in mouse RAW264.7 macrophages by Western blot**. LPS: 100 ng/mL. No LPS and the other drugs were added to the non-treatment group (NT). The experiment was duplicated. Original blots can be seen in supplement file “iNOS and Cox-2 original blots”.

The Western blot assay revealed that NAG > BNAG2>BNAG1 for inhibition of Cox-2 expression and BNAG1>BNAG2>NAG for inhibition of iNOS expression, respectively (Fig. 4).

**Fig. 5:**
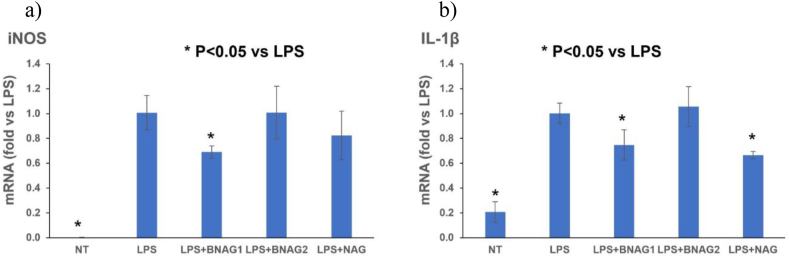
**The comparison of BNAG1, BNAG2, and NAG at 0.5 mM in iNOS (a) and IL-1β (b) expression in mouse RAW264.7 macrophages was determined by RT-PCR analysis.** LPS: 100 ng/mL. No LPS and the other drugs were added to the non-treatment group (NT). There were four repeats in each group (n = 4). *P < 0.05 vs LPS group. The experiment was duplicated.

**Fig. 6:**
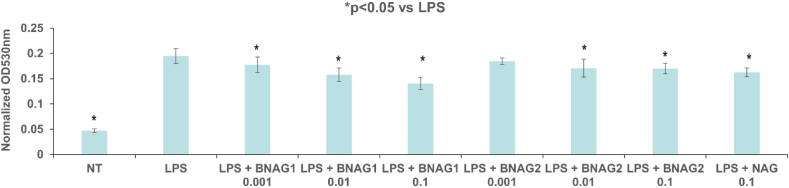
**The comparison of BNAG1, BNAG2, and NAG in nitrite production in LPS-activated mouse RAW264.7 macrophages.** Various concentrations (0.001 mM, 0.01 mM and 0.1 mM were tested for BNAG1 and BNAG2, while NAG at 0.1 mM was used as control. LPS: 100 ng/mL. No LPS and the other drugs were added to the non-treatment group (NT). There were six repeats in each group (n = 6). *P < 0.05 vs LPS group. The experiment was duplicated.

### 3.4 Quantitative RT-PCR analysis for cellular mRNA of iNOS and IL-1β

The RT-PCR showed that BNAG1 (0.5 mM) could inhibit mRNA expression of both iNOS and IL-1β (p < 0.05, n = 4), while NAG (0.5 mM) could inhibit IL-1β (p < 0.05, n = 4) but not iNOS mRNA expression (p > 0.05, n = 4). BNAG2 (0.5 mM) didn't inhibit either iNOS or IL-1β mRNA expression (p > 0.05, n = 4) (Fig. 5).

### 3.5 The Griess reagent test for nitrite production

The nitrite test showed that all three drugs tested (BNAG1, BNAG2, and NAG) could significantly inhibit NO production (n = 6), while BNAG1 was most effective among them. Notably, NO production in the presence of BNAG1 at 0.1 mM significantly lower than either BNAG2 or NAG at the same concentration, reflected by OD values of 0.141 ± 0.012 for BNAG1, 0.170 ± 0.010 for BNAG2 and 0.162 ± 0.009 for NAG, respectively (Fig. 6).

## 4 Discussion

In this study, we examined two newly designed compounds, BNAG 1 and 2, which are molecules obtained from modifications of NAG. Specifically, BNAG 1 was created from the removal of the hydroxyl groups at C1 and C3 of NAG. BNAG 2, on the other hand, was created from the removal of the hydroxyl groups at C4 and C6 of NAG (Fig. 1). We compared their anti-inflammatory properties to the parent molecule using an in vitro model of mouse macrophages RAW264.7 cells induced by LPS. We found that while all three compounds at the tested doses up to 5 mM demonstrated less than 10% inhibition against cell proliferation of RAW264.7 macrophage cells, except that BNAG 1 possessed a remarkable toxicity with its inhibition rate of 32.6% at 5 mM (Fig. 2). For IL-6 and TNF α production, BNAG 1 once again showed the highest potency with notable effects even at 0.01 mM (Fig. 3). Quantitative RT-PCR revealed that BNAG 1 and NAG inhibited the expression of IL-1β in LPS-induced inflammation of RAW 264.7 cells at 0.5 mM. BNAG 2, on the other hand, didn't show any suppression at this dose (Fig. 5). It was also found that while NAG and BNAG 2 showed inhibition of COX-2 expression at high and moderate levels respectively, BNAG 1 was barely noted to impact COX-2 on Western blot (Fig. 4). However, the results of Western blot and quantitative RT-PCR supported the superior effect of BNAG 1 on the expression of iNOS, a major enzyme producing NO under inflammatory conditions (Figs. 4 and 5). The Griess reagent test further supported BNAG1's impressive impact on the iNOS pathway by showing the highest reduction in NO production (Fig. 6).

There has long been a recognition that part of glucosamine metabolism includes its conversion to UDP-N-acetyl-glucosamine [19] and that N-acetyl-glucosamine can inhibit hexokinase, causing a potential ATP deficiency. Wolf et al. have noted that this interaction between NAG and hexokinase might lead to a downstream effect that is proinflammatory [20]. Given the current understanding of the anti-inflammatory effects of glucosamine and its derivatives, this is likely not the only pathway through which GlcN interacts with the immune system, as noted by the NFkB interaction described earlier. Nonetheless, NAG negatively interacts with hexokinase, which might be the cause of cytotoxicity. Since our molecules, BNAG 1 and 2, are derivatives from NAG, it seems entirely possible that this might also be the mechanism through which they exert their toxicity. The removal of the hydroxyl groups might have impacted the availability of these molecules in the cell, either through increased inward transportation, reduced breakdown, or decreased outward excretion. As such, they have been found to have stronger cytotoxicity compared to the parent molecule (Fig. 2). Notably, the removal of the hydroxyl groups on C1 and C3 in BNAG 1 seems to have resulted in a more dramatic effect (Fig. 2).

IL-1β, TNF α and IL-6 are extensively studied proinflammatory cytokines within the human immune system. They serve important physiological roles. However, they have also been implicated in a variety of pathologies with prominent inflammation components [21,22]. In the context of glucosamine and its derivatives, reduction of IL-1β, TNF α and IL-6 expression have historically been used to support modulation of the immune response. Shah et al. specifically, measured the serum levels of IL-1β and TNF α in adjuvant-induced arthritic model of rats to support the anti-inflammation efficacy of their molecule, N-[2,4,5-trihydroxy-6-(hydroxymethyl) tetrahydro-2Hpyran-3-yl]acrylamide (NHAG), an analog of glucosamine [23]. Shin et al. reported that another glucosamine analog, 2-deoxy-2-[(o-methylbenzylidene)]-β-glucopyranoside inhibited LPS-induced upregulation of IL-6, TNF α, and IL-1β in RAW264.7 cells [24]. Our results support the hypothesis that not only do BNAG 1 and 2 attenuate inflammation, but they also possess equivalent or even higher potency compared to the parent molecule (Figs. 3 and 5). Nonetheless, as IL-6, TNF α and IL-1β are relatively nonspecific markers of inflammation, they do not add much information regarding the mechanism of action.

Our evaluation of COX-2, iNOS, and NO, on the other hand, was designed to more closely investigate the possible downstream pathways involving BNAG 1 and BNAG 2. We already described earlier that glucosamine and its derivatives are believed to interfere with the inflammatory pathway in a multifaceted fashion, including through the NFkB pathway and the iNOS pathway [[15], [16], [17]]. Interestingly, BNAG 2 and especially BNAG 1 treatments exhibit a modest impact on the expression of COX-2 compared to NAG, which points to their lack of interaction with NFkB (Fig. 4). On the other hand, BNAG 1 was found to strongly mediate the expression of iNOS both qualitatively on Western blot and quantitatively on RT-PCR (Figs. 4 and 5). Downstream evaluation of the product using the Griess reagent also confirms BNAG 1's heavy involvement with nitrite production (Fig. 6). Taken together, these results suggest that the modifications we made to the N-acetyl-glucosamine molecule have increased its affinity to the iNOS pathway while reducing its ability to influence the NFkB pathway. This may prove to be beneficial given the ubiquity of the NFkB pathway and the possibility of unintended side effects. In conclusion, we propose that while these new molecules seem to influence different pathways, modifications at C1 and C3, in the case of BNAG 1, have caused this molecule to have the reduction of NO generation as the most prominent anti-inflammatory pathway of action. At the same time, the effect of C4 and C6 deoxygenation was much less pronounced which allowed BNAG 2 to be an intermediate molecule between BNAG 1 and NAG.

While we have been able to elucidate the anti-inflammatory effects of two new molecules, BNAG 1 and 2, there are several limitations to the study as well as areas needing further clarification. First, BNAG 1, the more efficacious molecule of the two, has been associated with cytotoxicity. Since BNAG 1's toxicity is toward RAW 264.7 mouse macrophages, a proinflammatory population of cells, it might contribute to BNAG 1's superior anti-inflammatory response. While we have proposed a possible explanation for this effect, namely the inhibition of hexokinase, further experiments must be pursued to confirm or deny this claim. The incorporation of BNAG 1 in a proinflammatory animal model would also be helpful to evaluate the effect of this cytotoxicity on inflammatory cells in vivo. Second, we found that the removal of hydroxyl groups from the original NAG molecule resulted in its reduced affinity to the NFkB but increased activity in the iNOS pathway. However, it is not clear why this is the case. An understanding of this would be useful to guide future modification of glucosamine and its derivatives. Third, our study is entirely in vitro. Inflammation was evaluated non-specifically using common markers including IL-1β and IL-6. This study would benefit from further characterization in an inflammatory animal model. Evaluation in vivo would provide the necessary information on whether BNAGs are viable strategies to target inflammation in a variety of pathologies, including but not restricted to osteoarthritis, rheumatoid arthritis, inflammatory bowel disease, and cancer.

## 5 Conclusions

In summary, we have been successful in showing that BNAG 1 and BNAG 2 possess the significant capacity to reduce expression in a variety of classic and specific inflammatory markers, superior to NAG (except for COX2). BNAG 1 specifically showed the strongest suppression of the expression or production of IL-6, IL-1β, TNF α, iNOS and NO. BNAG1 and BNAG 2 might be good candidates for a novel anti-inflammatory drug, further studies are recommended.

## Author contribution statement

Quang Le: Performed the experiments; Wrote the paper.

Zhichang Zhang; Daniel Sun: Performed the experiments.

Quanjun Cui; Ameer E. Hassan: Conceived and designed the experiments; Analyzed and interpreted the data.

Xinlin Yang: Conceived and designed the experiments; Performed the experiments; Analyzed and interpreted the data; Contributed reagents, materials, analysis tools or data; Wrote the paper.

## Data availability statement

Data will be made available on request.

## Declaration of interest’s statement

The authors declare no conflict of interest.

## Additional information

No additional information is available for this paper.

## Funding Statement

This work was supported by an unrestricted grant from Dr. Ameer E. Hassan to the gift fund of the Department of Orthopaedic Surgery, University of Virginia [104473-XY3C-DR00539-40916].
